# Ternary complex structures of human farnesyl pyrophosphate synthase bound with a novel inhibitor and secondary ligands provide insights into the molecular details of the enzyme’s active site closure

**DOI:** 10.1186/1472-6807-12-32

**Published:** 2012-12-12

**Authors:** Jaeok Park, Yih-Shyan Lin, Joris W De Schutter, Youla S Tsantrizos, Albert M Berghuis

**Affiliations:** 1 Department of Biochemistry, McGill University, 3649 Promenade Sir William Osler, Montreal, QC, Canada; 2 Department of Microbiology and Immunology, McGill University, 3649 Promenade Sir William Osler, Montreal, QC, Canada; 3 Groupe de Recherche Axé sur la Structure des Protéines, McGill University, 3649 Promenade Sir William Osler, Montreal, QC, Canada; 4 Department of Chemistry, McGill University, 801 Sherbrooke Street West, Montreal, QC, Canada

**Keywords:** Mevalonate pathway, Farnesyl pyrophosphate synthase, Isopentenyl pyrophosphate, Bisphosphonates, C-terminal tail closure, Cancer chemotherapeutics

## Abstract

**Background:**

Human farnesyl pyrophosphate synthase (FPPS) controls intracellular levels of farnesyl pyrophosphate, which is essential for various biological processes. Bisphosphonate inhibitors of human FPPS are valuable therapeutics for the treatment of bone-resorption disorders and have also demonstrated efficacy in multiple tumor types. Inhibition of human FPPS by bisphosphonates in vivo is thought to involve closing of the enzyme’s C-terminal tail induced by the binding of the second substrate isopentenyl pyrophosphate (IPP). This conformational change, which occurs through a yet unclear mechanism, seals off the enzyme’s active site from the solvent environment and is essential for catalysis. The crystal structure of human FPPS in complex with a novel bisphosphonate YS0470 and in the absence of a second substrate showed partial ordering of the tail in the closed conformation.

**Results:**

We have determined crystal structures of human FPPS in ternary complex with YS0470 and the secondary ligands inorganic phosphate (Pi), inorganic pyrophosphate (PPi), and IPP. Binding of PPi or IPP to the enzyme-inhibitor complex, but not that of Pi, resulted in full ordering of the C-terminal tail, which is most notably characterized by the anchoring of the R351 side chain to the main frame of the enzyme. Isothermal titration calorimetry experiments demonstrated that PPi binds more tightly to the enzyme-inhibitor complex than IPP, and differential scanning fluorometry experiments confirmed that Pi binding does not induce the tail ordering. Structure analysis identified a cascade of conformational changes required for the C-terminal tail rigidification involving Y349, F238, and Q242. The residues K57 and N59 upon PPi/IPP binding undergo subtler conformational changes, which may initiate this cascade.

**Conclusions:**

In human FPPS, Y349 functions as a safety switch that prevents any futile C-terminal closure and is locked in the “off” position in the absence of bound IPP. Q242 plays the role of a gatekeeper and directly controls the anchoring of R351 side chain. The interactions between the residues K57 and N59 and those upstream and downstream of Y349 are likely responsible for the switch activation. The findings of this study can be exploited for structure-guided optimization of existing inhibitors as well as development of new pharmacophores.

## Background

The mevalonate pathway produces essential lipid molecules, such as steroids and isoprenoids, in mammalian cells. Occupying the first branching point of the mevalonate pathway, farnesyl pyrophosphate synthase (FPPS) catalyzes the sequential elongation of dimethylallyl pyrophosphate (DMAPP) to geranyl pyrophosphate (GPP) and then to farnesyl pyrophosphate (FPP) via successive condensation of two isopentenyl pyrophosphate (IPP) molecules. The subsequent condensation of FPP and another IPP unit in the pathway produces geranylgeranyl pyrophosphate (GGPP). Covalent attachment of FPP and GGPP (i.e. prenylation) is critical for the proper subcellular localization and function of many proteins, including small GTPases that regulate a wide variety of cellular processes [1]. FPPS, therefore, is an attractive point of pharmacological intervention. Bisphosphonate inhibitors of FPPS, for instance, are widely used to treat a number of bone disorders, such as Paget’s disease, hypercalcemia, metastatic osteolysis, and osteoporosis currently [2]. Given the crucial roles of small GTPases in cancer development [3-5], the ability of bisphosphonates to inhibit FPPS and consequently the prenylation of these proteins qualifies them also as potential cancer chemotherapeutics. In addition, FPPS inhibition produces a secondary anticancer effect via the accumulation of IPP, which activates human γδ T immune cells [6,7]. Recent clinical studies have demonstrated that bisphosphonate drugs enhance the antitumor effects of various existing therapeutics synergistically and improve survival in patients with prostate cancer, breast cancer, and multiple myeloma [8].

The mechanism by which bisphosphonates inhibit human FPPS has been examined through characterization of X-ray crystal structures. These compounds bind at the DMAPP/GPP sub-pocket of the enzyme’s active site, mimicking and competing against these substrates [9,10]. The enzyme inhibition also involves a sequence of conformational changes [9,10]. Bisphosphonate binding to the “open” form of the enzyme drives a rigid body movement that closes the entrance to the DMAPP/GPP sub-pocket. This conformational change fully shapes the second substrate binding site, the IPP sub-pocket. Subsequent IPP binding to the now “partially closed” enzyme induces the closing of the ^350^KRRK^353^ C-terminal tail, which is disordered in the absence of bound IPP, over the IPP entry site. These C-terminal basic residues are essential for catalysis [11], and upon closing secure the ligands into position and sequester the catalytic cavity from water. With the enzyme in this “fully closed” state, replacement of the deeply buried bisphosphonate inhibitor by a competing substrate is very difficult, and hence the binding of the bisphosphonate is deemed nearly irreversible. The exceptional in vivo efficacy of bisphosphonate drugs, therefore, is thought to arise in part from the stabilization of the enzyme-inhibitor complex by the binding of the accumulating substrate IPP [10]. Despite its importance, however, the molecular details responsible for the tail closure in human FPPS are largely uncharacterized. That this conformational change is induced by IPP is especially intriguing, as the ligand does not make any direct contact with the ^350^KRRK^353^ tail when bound to the enzyme.

**Figure 1:**
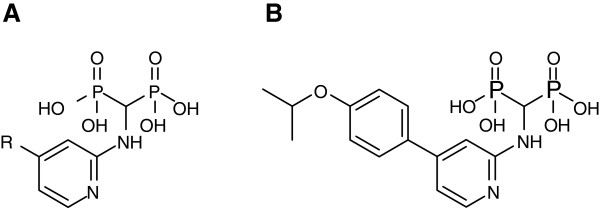
**Novel bisphosphonate inhibitors of human FPPS.** (**A**) General structure of 2-aminopyridine-based bisphosphonate inhibitors (see ref. [12] for the complete list of functional groups at the substitution site). (**B**) Compound YS0470.

The bisphosphonates in current clinical use, such as risedronate and zoledronate, are highly hydrophilic. The resultant low membrane permeability, as well as their extreme affinity for bone tissue, compromises the full antineoplastic potential of these drugs. In an effort to develop FPPS inhibitors with superior physicochemical properties, we have recently identified potent and selective 2-aminopyridine-based bisphosphonates (Figure 1A) [12]. The crystal structure [PDB: 4DEM] of human FPPS in complex with one of our new inhibitors, YS0470 (Figure 1B), revealed a series of novel interactions and conformations in and around the active site cavity [12]. Most interestingly, the ^350^KRRK^353^ tail of the enzyme was partially ordered, with its main chain covering over the IPP sub-pocket but its side chains fully flexible [12]. This finding provided the first report of such a conformational state in human FPPS and raised a question whether the IPP sub-pocket in the enzyme-YS0470 complex is still accessible to a ligand. In order to address this question, we have determined crystal structures of human FPPS in ternary complex with YS0470 and the secondary ligands inorganic phosphate (Pi), inorganic pyrophosphate (PPi), and IPP. Prior to the present communication there have been only two IPP-bound human FPPS structures available, determined independently but both co-bound with zoledronate [9,10], and none with bound PPi. Crystal structure analysis and complementary isothermal titration calorimetry (ITC) and differential scanning fluorometry (DSF) experiments reveal new insights into the details of the ligand-induced conformational changes in human FPPS and suggest a mechanism for the enzyme’s C-terminal tail closure. A better understanding of the human FPPS tail closure may help develop new classes of anticancer drugs that function by targeting this crucial conformational change.

## Methods

### Crystal-soaking and data collection

Expression and purification of recombinant human FPPS, as well as crystallization of the enzyme-YS0470 binary complex, were carried out as previously described [12]. Aqueous solutions of Pi and PPi were prepared at 5 mM concentration. IPP was prepared at 4.5 mM concentration in 1 M Tris buffer. For each ligand, 0.4 μL of the stock solution was added to a 2 μL hanging drop containing two to three single crystals. The coverslips holding the ligand-added drops were resealed over the same reservoir wells in the original crystallization tray, which was then incubated overnight to ensure maximal diffusion of the ligand into the crystals. Diffraction data were collected from a single crystal for each ligand under a nitrogen cryo-stream as 0.5° oscillation images by using a Rigaku RUH3R X-ray generator with a rotating Copper anode and a Rigaku R-AXIS IV++ area detector.

### Data processing and structure refinement

The diffraction images were processed with HKL2000 [13]. The initial structures were determined by difference Fourier methods with the ligand-omitted structure of the human FPPS-YS0470 binary complex [PDB: 4DEM] as an input model. These structures were further improved through iterative rounds of manual and automated refinement with COOT [14], PHENIX [15], and REFMAC5 [16]. The final models of the ternary complexes have been deposited into the Protein Data Bank. Data collection and refinement statistics, as well as the PDB ID for each structure, is presented in Table 1.

**Table 1:** Data collection and refinement statistics

**Data sets**			
PDB ID	4H5C	4H5D	4H5E
Ligands	YS0470, Pi	YS0470, PPi	YS0470, IPP
**Data collection**			
Space group	*P*4_1_2_1_2	*P*4_1_2_1_2	*P*4_1_2_1_2
Unit cell dimension (Å)	*a* = *b* = 110.98, *c* = 66.79	*a* = *b* = 111.53, *c* = 66.24	*a* = *b* = 111.24, *c* = 65.65
Resolution range (Å)	50.0-2.02 (2.05-2.02)	50.0-2.02 (2.05-2.02)	50.0-2.05 (2.09-2.05)
Redundancy	26.4 (24.1)	28.2 (27.9)	27.3 (26.2)
Completeness (%)	99.9 (100)	100 (100)	100 (100)
*I*/*σ*(*I*)	36.9 (5.7)	59.9 (7.7)	47.7 (6.9)
*R*_merge_	0.061 (0.475)	0.043 (0.418)	0.043 (0.369)
**Refinement**			
No. reflections	26,374	26,414	24,849
*R*_work_/*R*_free_	0.180/0.213	0.180/0.203	0.186/0.233
No. protein atoms	2,705	2,748	2,743
No. ligand atoms	31	35	40
No. ion atoms	3	3	3
No. solvent atoms	132	136	108
**R.m.s. deviations**			
Bond length (Å)	0.018	0.019	0.020
Bond angle (°)	1.8	1.9	2.0

### Structure analysis

Structure analysis and model representation were performed with the molecular modeling and visualization programs COOT [14] and PyMOL (version 1.3 Schrödinger, LLC.). Information on the structure models used in this analysis, including the PDB IDs and bound ligands, is summarized in Table 2.

**Table 2:** Human FPPS structure models analyzed in this study

**PDB ID**	**Res. (Å)**	**Bisphosphonate inhibitor**^**a**^	**Other ligands**	**Conformational state**	**Literature reference**
2F7M	2.30	-	-	Open	[10]
2F8C	2.20	Zoledronate	Pi	Partially closed	[10]
2F8Z	2.60	Zoledronate	IPP	Fully closed	[10]
1YV5	2.00	Risedronate	Pi	Partially closed	[9]
1ZW5	2.30	Zoledronate	IPP	Fully closed	[9]
3N45	1.88	Zoledronate	Pi, FBS_04^b^	Partially closed	[21]
3N46	2.35	Zoledronate	Pi, NOV_980^b^	Partially closed	[21]
4DEM	1.85	YS0470	-	Partially closed	[12]
4H5C	2.02	YS0470	Pi	Partially closed	Current report
4H5D	2.02	YS0470	PPi	Fully closed	Current report
4H5E	2.05	YS0470	IPP	Fully closed	Current report

^a^All bisphosphonates are bound with Mg^2+^ ions.

^b^Allosteric inhibitors.

Please note that in the PDB structures 1YV5, 1ZW5, and 4DEM, the amino acids are numbered with an offset of 14 compared to the rest of the structures. The C-terminal tail KRRK residues in these structures are thus numbered from 364 to 367.

### Isothermal titration calorimetry

ITC experiments were performed with a VP-ITC titration calorimeter from GE Healthcare. The human FPPS sample was prepared at 50 μM concentration in the presence of 150 μM YS0470 and 200 μM MgCl_2_ in a buffer containing 10 mM HEPES (pH 7.5), 500 mM NaCl, 2 mM β-mercaptoethanol, and 5% glycerol. Ligand solutions were prepared in the same buffer at concentrations ranging from 0.1 to 1 mM. Titrations were carried out at 30 °C, and the data were analyzed with the Origin 7 software provided by the manufacturer.

### Differential scanning fluorometry

The samples were prepared to the final volume of 40 μL in the following concentrations: 4 μM protein, 10 mM HEPES (pH 7.5), 10 mM NaCl, 5 mM MgCl_2_, and 5× SYPRO Orange dye (Invitrogen, commercial stock solution is 5000×). When added, the final concentration of YS0470 was 40 μM, and those of the secondary ligands (Pi, PPi, and IPP) were up to 400 μM. All samples were prepared in triplicate. Fluorescence was measured by using an iCycler RT-PCR instrument with an iQ5 detector (Bio-Rad) while heating the samples in a gradient from 30 to 90°C in 0.5°C steps every 10 s. The midpoint temperature of the unfolding transition (*T*_*m*_) was calculated by using the software package Bio-Rad iQ5.

## Results and discussion

### Structures of the human FPPS ternary complexes

We were able to obtain crystals of human FPPS in ternary complex with different ligands. X-ray diffraction analysis of these crystals has clearly identified the secondary ligands (i.e. Pi, PPi, and IPP) bound in the IPP sub-pocket, as well as the inhibitor YS0470 in the DMAPP/GPP sub-pocket. Co-binding of the secondary ligands did not affect the pre-bound inhibitor, as the binding location and orientation of this inhibitor, as well as its interaction with the enzyme, remain unchanged in the three ternary complexes from those previously observed in the binary complex [PDB: 4DEM] [12].

**Figure 2:**
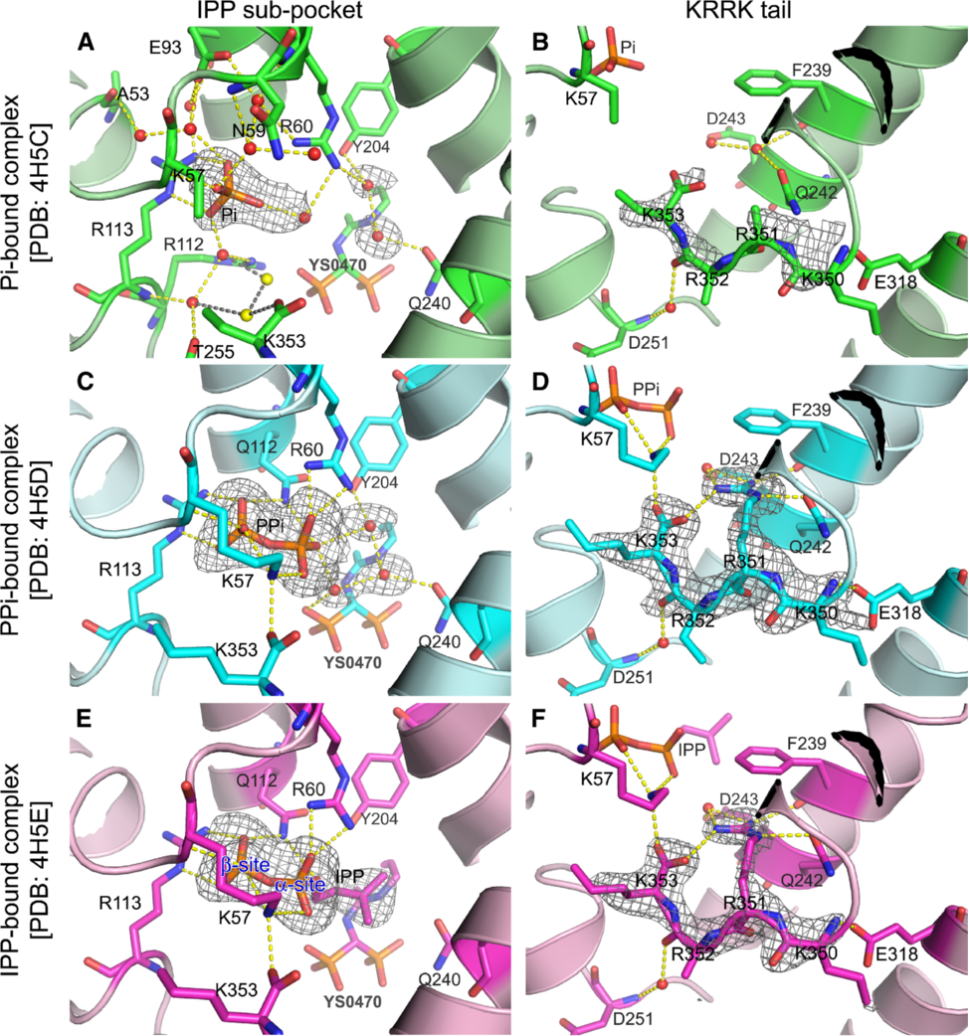
**Structures of human FPPS ternary complexes at the IPP sub-pocket and the **^**350**^**KRRK**^**353**^** tail regions.** (**A**) The cavity of the IPP sub-pocket is filled with ordered water molecules as well as the bound Pi. The water molecules, which are displayed as red spheres, neutralize the surrounding charged residues and also provide structural support via hydrogen bonds (yellow dashes) to this flexible region. The yellow spheres represent the two water molecules present in the FPPS-YS0470 binary complex [PDB: 4DEM] but not in the FPPS-YS0470-Pi ternary complex, and the grey dashes show their interactions with the adjacent entities. (**B**) The ^350^KRRK^353^ tail is in the same conformation as that previously seen in the FPPS-YS0470 binary complex. Compound YS0470 and Pi are bound deeper in the active site and too far from the C-terminal region to have any direct interaction with the tail residues. (**C** and **E**) The phosphate groups of the bound PPi and IPP superpose very well. Other bound water molecules and water-mediated hydrogen bonding interactions are omitted for clarity. We have sub-divided the PPi binding site into the alpha- and beta-sites for easier description. (**D** and **F**) The ^350^KRRK^353^ tail is in the fully closed conformation in the both complexes. The grey meshes represent simulated-annealing omit (*F*_o_-*F*_c_) maps contoured at 3.0 sigma level. Carbon atoms are represented in green, cyan, and magenta, for the FPPS-YS0470-Pi, FPPS-YS0470-PPi, and FPPS-YS0470-IPP ternary complexes, respectively. The color schemes for other atoms (red for oxygen; blue for nitrogen; and orange for phosphorous) are consistent throughout all the figures.

The overall structure of the human FPPS-YS0470-Pi ternary complex is very similar to that of the binary complex, with the backbone RMSD value of 0.19 Å. The Pi molecule, found at the previously described IPP binding site [9,10], forms direct polar contacts with the main chain of K57 and the side chain of R113 (Figure 2A). In addition, the ligand is further stabilized by an intricate network of water-mediated hydrogen bonds involving a number of residues including N59, R60, E93, and R112 (Figure 2A). The space occupied by Pi in the ternary complex instead accommodates a water molecule with a high B-factor in the binary complex. Another difference between the two structures is the absence of two ordered water molecules (shown as yellow spheres in Figure 2A) near the C-terminal residue K353 in the ternary complex. The reason for this discrepancy is unclear, but these water molecules may contribute to the closing of the ^350^KRRK^353^ tail by bridging hydrogen bond interactions between K353 and the residues R112, R113, and T255 (Figure 2A).

The ^350^KRRK^353^ tail of the Pi-bound ternary complex is similar to that of the binary complex, with flexible and thus undefined side chains (Figure 2B). Although the backbone atoms of the tail could be modeled in and refined without much difficulty during the structure refinement process, the electron density around this area was weak (Figure 2B), suggesting partial ordering. A direct hydrogen bond between the side chain of E318 and the main chain nitrogen of K350, and a water-mediated hydrogen bond between the main chain nitrogen of D251 and the main chain oxygen of R352, may be responsible for the partial ordering of the tail at this location (Figure 2B). The water molecule mediating the latter hydrogen bonding interaction is well ordered, as also seen in the binary complex previously [12]. The side chain nitrogen of Q242 is within the hydrogen bonding range to the main chain oxygen of R351 (Figure 2B), but such an interaction is not likely, as the angle between the hydrogen donor and acceptor atoms is suboptimal in the current conformational state.

The overall structures of the PPi- and IPP-bound ternary complexes are more similar to each other (RMSD: 0.16 Å) than to those of the binary complex and the Pi-bound ternary complex (RMSD: 0.25-0.31 Å). The PPi and IPP-binding sites fully overlap the Pi-binding site, which we refer to as the beta phosphate site (Figure 2A, C, and E). The space that accommodates the alpha phosphate moiety of IPP, or the alpha phosphate site (Figure 2E), is instead occupied by a single water molecule in both the Pi-bound ternary complex (Figure 2A) and the binary complex [PDB: 4DEM]. The phosphate moiety at the alpha site provides additional charge interactions with the residues R60 and Q112 in both the PPi- and IPP-bound complexes (Figure 2C and E). The isopentenyl tail of the bound IPP ligand extends toward the bisphosphonate inhibitor YS0470 (Figure 2E), displacing water molecules otherwise present in the area (Figure 2A and C). These water molecules in the PPi-bound complex form a tight set of hydrogen bonds connecting the PPi and bisphosphonate molecules and the residues Y204 and Q240 (Figure 2C). Other water molecules and water-mediated interactions around the secondary ligands in the PPi- and IPP-bound complexes are analogous to those in the Pi-bound ternary complex and the binary complex.

The ^350^KRRK^353^ tail in both the PPi- and IPP-bound ternary complexes is closed and fully ordered, as evidenced by the clearly defined electron density (Figure 2D and F), as well as the average B-factors of the four C-terminal residues (Additional file 1: Table S1). This conformational state is most notably characterized by the rigidification of R351 (Figure 2D and F), as also observed in the human FPPS-zoledronate-IPP ternary complex [PDB: 1ZW5 and 2F8Z] previously [9,10]. All three side chain nitrogen atoms of R351 are involved in either direct or water-mediated hydrogen bonding to the neighbouring residues F239, Q242, D243, and K353, thereby holding the tail tightly in position (Figure 2D and F). The side chain of K57 in the PPi- and IPP-bound complexes is also more ordered compared to that of the Pi-bound complex, forming hydrogen bonds to the secondary ligand PPi or IPP and the terminal oxygen atom of K353 (Figure 2B, D, and F). The structures of our PPi- and IPP-bound enzyme complexes thus represent the “fully closed” state, in contrast to the structure of our “partially closed” Pi-bound complex (summarized in Table 2).

### Binding of Pi, PPi, and IPP to the human FPPS-YS0470 binary complex

In this work, we have shown for the first time that the binding of PPi induces the full closure of the ^350^KRRK^353^ tail in human FPPS. This finding is interesting because PPi, unlike IPP, is not a substrate for the enzyme. The PPi molecule in our ternary complex does not represent a product state either, although the FPPS reaction produces PPi, as it is in the IPP sub-pocket. The transfer of the allylic chain in this reaction, which is from DMAPP or GPP to IPP [17,18], should leave the product PPi in the DMAPP/GPP sub-pocket. The PPi-induced C-terminal tail closure also demonstrates that the presence of the isopentenyl moiety on the secondary ligand is not a necessary condition for this conformational change in the human FPPS ternary complex. Rather, it is the phosphate moiety occupying the alpha phosphate site that is decisively responsible for the tail closure of the protein. The structure of our Pi-bound ternary complex, as well as those previously determined by others (Table 2), indicates that the occupancy of the beta phosphate site by a single phosphate group alone cannot induce this conformational change.

**Figure 3:**
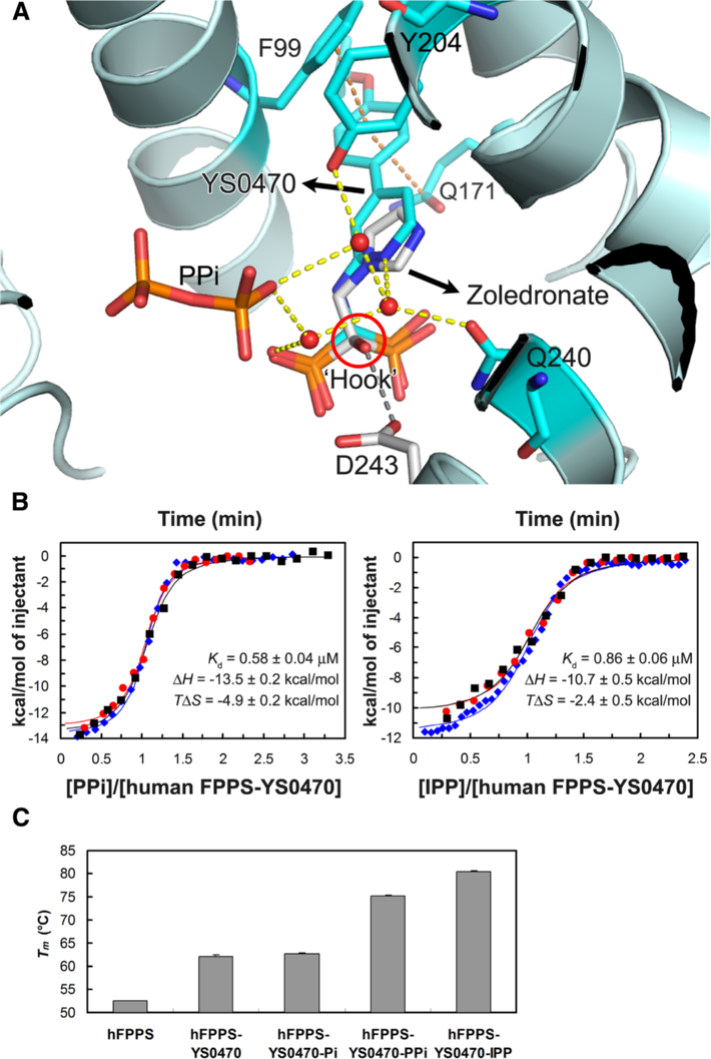
**Ligand binding to human FPPS.** (**A**) The active site structures of the FPPS-YS0470-PPi complex (cyan) and the FPPS-zoledronate-IPP complex (white) [PDB: 1ZW5] are presented superimposed. The IPP molecule in the zoledronate complex is not shown for clear visualization of the bisphosphonates. The hydroxyl ‘hook’ of the bound zoledronate is circled in red. This functional group forms a hydrogen bond with the side chain of D243, which is represented as a grey dashed line in the illustration. The orange dashed lines represent stacking interactions between YS0470 and the residues F99 and Q171. (**B**) ITC isotherms for the binding of PPi and IPP to the human FPPS-YS0470 complex. Three independent experiments were carried out for each ligand. The binding parameters were determined from each data set by using a one-site model. The reported values are Mean ± SEM from the three experiments. *T* = 303.15 kelvin. (**C**) A bar graph showing melting temperatures of human FPPS in various ligand-bound states measured by DSF. In order to ensure complete complex formation, YS0470 and the secondary ligands were added at 10- and 100-fold molar excess of the protein, respectively.

A crucial implication of the PPi-induced C-terminal tail closure in human FPPS is that PPi, which is a prevalent cellular metabolite, may have a relevant role in the inhibition of the enzyme in vivo. Based on the structures of our PPi and IPP-bound ternary complexes, which are both in the fully closed state, PPi would at least be equally effective as IPP in stabilizing the protein-inhibitor complex, if not better. The inner surface of the IPP sub-pocket of FPPS is highly hydrophilic, and the hydrophobic isopentenyl tail of IPP is forced to stack against a number of hydrophilic side chain functional groups upon its binding to the enzyme. These interactions may be beneficial for the catalysis and the subsequent release of the product in the isoprenoid elongation reaction, but not for stabilizing the enzyme-bisphosphonate complex in the closed state. With PPi binding, on the other hand, the water molecules bound in place of the isopentenyl tail of IPP may further stabilize the protein-inhibitor complex by linking the ligands and the surrounding residues. These water molecules play an especially important role in the binding of our inhibitor YS0470, which lacks the hydroxyl ‘hook’ present in most of the currently used bisphosphonate drugs, by making up for the missing polar interaction seen in the presence of this functional group (Figure 3A). The absence of this hydroxyl side chain in our series of bisphosphonates is advantageous for the purpose of targeting non-bone tissues, because the presence of this substituent in a bisphosphonate increases its affinity for bone mineral [19,20].

In order to confirm that PPi indeed forms a tighter complex with human FPPS and YS0470 than IPP, we have carried out ITC experiments in which the secondary ligands were titrated into a protein sample pre-saturated with the inhibitor and magnesium ions. The dissociation constant (*K*_d_) was determined to be lower for PPi (Figure 3B), indicating that PPi-binding is indeed stronger than IPP-binding. The higher binding affinity of PPi is due to a more favorable (more negative) enthalpy change (Δ*H*, Figure 3B), which corroborates our prediction that the strength of interactions between the ligand and the protein-inhibitor complex is greater for PPi. The entropic component of binding (*T*Δ*S*), on the other hand, was shown to be more favorable (less negative) for IPP (Figure 3B), although not sufficient to fully counter the enthalpic effect. The difference in the entropy change is likely due to desolvation effects: more water molecules would be released from the binding site and the ligand itself upon IPP-binding, resulting in greater degrees of freedom in the system. In contrast to the solvation entropy change, the conformational entropy change should be slightly less favorable for IPP binding, as IPP with more rotatable bonds within the molecule than PPi would lose greater degrees of conformational freedom upon binding. Changes in protein conformational entropy resulting from ligand binding should be similar between PPi and IPP, based on our structural data.

We have also examined the binding of Pi to the human FPPS-YS0470 complex by ITC, but could not reliably determine its energetic profile due to a low heat signal. The weak thermodynamic signature likely reflects low affinity binding, as well as the absence of a major conformational change (e.g., the C-terminal tail closure) induced by the binding. Complimentary DSF experiments indeed confirmed the lack of any significant conformational change induced by Pi binding. As seen in Figure 3C, the *T*_*m*_ of human FPPS increases ~10°C in the presence of YS0470, indicating that the enzyme is more thermally stable in its partially closed state than in the open state. Addition of the secondary ligands PPi and IPP further stabilizes the enzyme, likely via the full closure of the enzyme, whereas Pi does not provide any additional thermal protection, indicating the lack thereof. It is interesting here that the human FPPS complex shows a higher *T*_*m*_ in the presence of IPP (80°C) than with PPi (75°C). These values are seemingly at odds with the results of the ITC experiments, suggesting that IPP forms a tighter complex with human FPPS and YS0470 than PPi. However, as described earlier, PPi binding results in a more favorable enthalpy change (Δ*H*) than IPP binding but a less favorable entropy change (Δ*S*). The entropic effects of course become increasingly pronounced for ligand binding as the temperature rises. Based on the Δ*H* and Δ*S* values determined from the ITC experiments (Figure 3B), the binding of IPP to the human FPPS-YS0470 complex becomes more favorable than that of PPi only at temperatures above ~70°C.

### Mechanistic details of the C-terminal tail closure in human FPPS

As mentioned previously, the molecular details responsible for the tail closing action in human FPPS are largely unknown, despite its functional importance. What is clear, however, is that the role of the R351 side chain is absolutely critical in the full closing of the ^350^KRRK^353^ tail. This side chain not only anchors the residue itself to the ^221^G-E^247^ helix, one of the longest central helices of human FPPS, but also helps hold the last residue K353 in position by providing a salt bridge (as seen in Figure 2D and F). The electron density observed for our Pi-bound complex has demonstrated that the side chain of R351 can still be entirely flexible, while the main chain of the C-terminal tail is partially ordered and structured (as seen in Figure 2B). This finding suggests that the recruitment of the tail to the approximate region occurs first, where the tail is held loosely by other interactions perhaps involving those described earlier (Figure 2A and B), prior to the rigidification of the R351 side chain.

**Figure 4:**
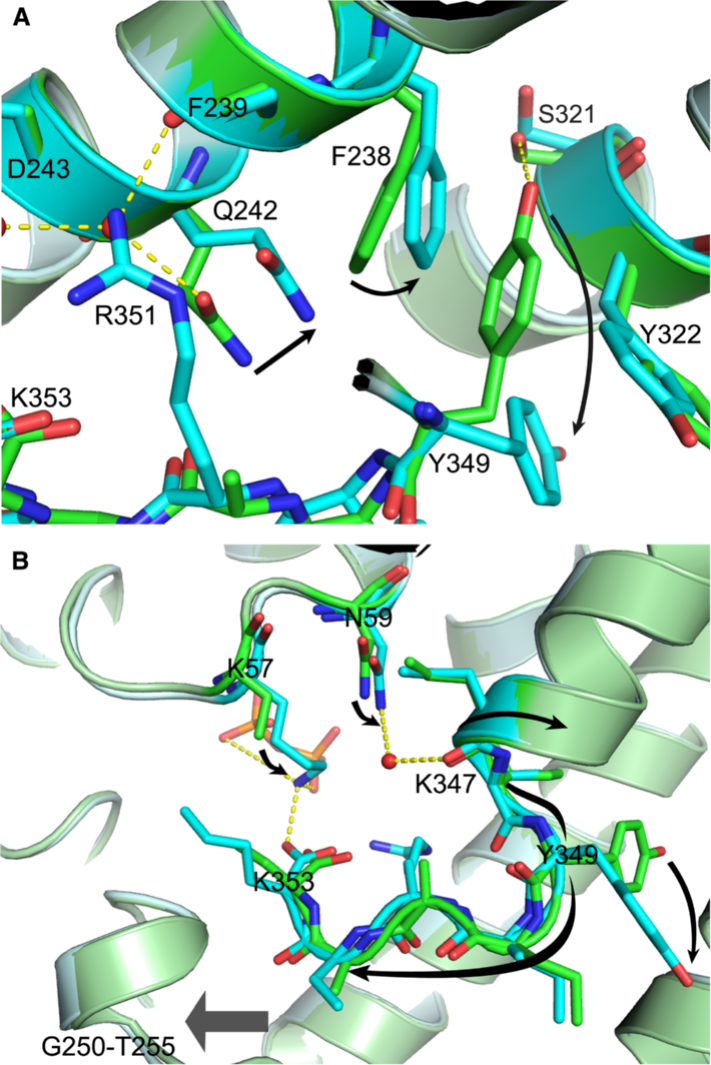
**Residues involved in the human FPPS C-terminal tail closure.** (**A**) The structures of the FPPS-YS0470-Pi (green) and FPPS-YS0470-PPi (cyan) complexes are superimposed. The conformational changes that occur prior to the rigidification of the R351 side chain are indicated with black arrows. The residues Y349, F238, and Q242 are in the anchor-blocking conformation in the Pi-bound complex and in the anchor-accepting conformation in the PPi-bound complex. (**B**) A schematic representation of the Y349 switch activation: the K57 side chain rigidifies and attracts the C-terminal tail; N59 interacts with K347 via a water molecule; and the Y349 side chain rotates out due to the torsion created by these two forces.

Analysis of our FPPS structures suggests that proper positioning and ordering of the R351 side chain also requires a series of preceding conformational changes in the residues Q242, F238, and Y349. In the absence of bound PPi/IPP, Q242 forms a hydrogen bond to a nearby water molecule and together with it blocks the anchoring of the R351 side chain to the ^221^G-E^247^ helix (Figure 4A). The conformational change in Q242, in turn, requires a ~20° rotational translocation of the F238 side chain, which is prohibited due to steric hindrance by the Y349 side chain in the absence of PPi/IPP (Figure 4A). In this anchor-blocking conformation, the Y349 side chain is stacked tightly in position between the side chains of F238 and Y322, and is further stabilized via a polar interaction with the residue S321 (Figure 4A). In the anchor-accepting conformation, on the other hand, the side chain of Y349, as well as those of the adjacent aromatic residues F238 and Y322, has significantly greater freedom of movement, as evident from the electron density maps and the refined B-factors (Additional file 2: Figure S1). The above findings suggest that Y349, lying upstream in the cascade of these conformational changes, functions as a safety switch, which is normally locked in the “off” mode to prevent any futile C-terminal tail closure. Q242, on the other hand, plays the role of a gatekeeper in the enzyme, which directly controls the anchoring of R351. The greater structural freedom of the three aromatic residues (i.e. F238, Y322, and Y349) in the fully closed form of the enzyme may contribute to compensate for the reduction in conformational entropy caused by the ordering of the tail.

Despite the many currently available FPPS structures, it is still unclear how PPi/IPP binding turns on the Y349 switch in the human enzyme. This process is particularly intriguing, as the binding site for the secondary ligands is quite far (> 10 Å) from the tyrosine residue, whose conformational change is yet very drastic (i.e. ~80° rotation of the side chain). Comparison of the new ternary structures has allowed us to propose the following putative mechanism. Simultaneous occupancy of the alpha and beta phosphate sites by a pyrophosphate group in the IPP sub-pocket rigidifies the otherwise flexible K57 side chain (Figure 4B). The side chain of K57 in turn attracts the ^350^KRRK^353^ tail, which is partially present and/or structured in the vicinity, by forming a salt bridge with the terminal carboxyl group (Figure 4B). This ~0.7 Å shift of the tail is not likely a result of a modeling artefact, as it is accompanied by a similar movement in the adjacent ^250^G-T^255^ helix-turn segment (Figure 4B), of which such a shift induced by IPP binding has been also observed in the zoledronate-bound enzyme complexes [PDB: 2F8C, 2F8Z, and 1ZW5]. In addition to the rigidification of the K57 side chain, PPi/IPP binding results in a ~10° angular shift of the N59 side chain, which is translated into a similar movement of the residues K347 and I348 via a water molecule (Figure 4B). Interestingly, no water molecule has ever been observed at this position in human FPPS, except in the presence of bound PPi or IPP. These PPi/IPP-induced interactions between K57 and the residues downstream of Y349, and between N59 and those immediately upstream, may create a torque strong enough to rotate the Y349 side chain out of its “off” conformation (Figure 4B).

### Implications of the C-terminal tail closure on drug development

The activity of human FPPS involves a dynamic behavior, in which the enzyme alternates between the substrate-binding (open/partially closed) and catalytic (fully closed) states. As biasing this equilibrium either way results in enzyme inhibition, compounds that can inhibit the enzyme’s C-terminal tail closure can also inhibit its function. The non-bisphosphonate inhibitors of human FPPS identified in a recent fragment-based screening study [21] are of much relevance. These inhibitors, which act by binding to an allosteric site adjacent to the IPP sub-pocket, have completely different chemical scaffolds from the existing bisphosphonate drugs and thus a great potential as chemotherapeutic agents to target non-bone tissues. The mechanism of inhibition by this series of compounds is thought to involve a carboxylic functional group that interferes with the binding of IPP by electrostatic repulsion [21]. In addition, some of these compounds that are bulkier (e.g., FBS_04 and NOV_980, in the PDB structures 3N45 and 3N46, respectively) directly inhibit the closing of the C-terminal tail by steric hindrance [21]. Intriguingly, the location of the newly identified allosteric pocket is such that N59 and K347, which mediate the communication between the IPP sub-pocket and the tyrosine switch according to our proposed mechanism (as seen in Figure 4B), form opposite walls of this pocket. We predict that, therefore, compounds which occupy the allosteric pocket of human FPPS would inhibit its tail closure even in the presence of bound IPP, by disrupting the interaction between N59 and K347. Based on this prediction, then, elimination of the carboxylic group from the aforementioned allosteric inhibitors should not affect their ability to inhibit the enzyme, although their binding affinity would likely be changed. Removal of this highly charged moiety or its replacement with a preferable functional substituent may improve the pharmacokinetic properties of these compounds, proving the usefulness of the novel insights regarding the human FPPS tail closure provided in the present communication. Alternatively, by taking advantage of the new information, it may be possible to develop inhibitors that can trigger the C-terminal tail closure without IPP and lock the enzyme in the fully closed state, as the concept for such inhibitors was proposed recently [22].

## Conclusions

We have previously shown that the ^350^KRRK^353^ tail in the human FPPS-YS0470 binary complex is only partially structured. Here we have demonstrated that the binding of PPi or IPP to the IPP sub-pocket of the binary complex, but not that of Pi, fully structures the enzyme’s tail, which seals off the active site from the solvent environment and thereby stabilizes the protein-inhibitor complex. By examining the human FPPS structures presented in this report and those previously determined by others, we have also identified key residues and interactions responsible for this C-terminal rigidification. The tail closure is controlled by a safety mechanism involving the residue Y349, which is trapped in the “off” conformation in the absence of bound PPi/IPP. Turning on the tyrosine switch allows the gatekeeper Q242 to take on the anchor-accepting conformation, which in turn results in the clamping of R351 to the enzyme’s main frame. The rigidification of the anchor arginine is likely the final step in the sequence of events required for the complete closure of the ^350^KRRK^353^ tail. The process by which PPi/IPP-binding activates the tyrosine switch is not entirely clear, but we speculate that it involves subtle rotations and shifts initiated in the residues around the IPP sub-pocket and transmitted to those directly preceding and succeeding the tyrosine switch. The structures of the new ternary complexes of human FPPS will prove useful for optimizing clinically relevant inhibitors. Further, the novel insights with respect to the enzyme’s tail closing mechanism may help develop entirely new pharmacophores.

## Abbreviations

FPPS: Farnesyl pyrophosphate synthase; DMAPP: Dimethylallyl pyrophosphate; GPP: Geranyl pyrophosphate; FPP: Farnesyl pyrophosphate; IPP: Isopentenyl pyrophosphate; GGPP: Geranylgeranyl pyrophosphate; Pi: Inorganic phosphate; PPi: Inorganic pyrophosphate; ITC: Isothermal titration calorimetry; DSF: Differential scanning fluorometry; RMSD: Root-mean-square deviation.

## Competing interests

The authors declare that they have no competing interests.

## Authors’ contributions

JP conceived of and carried out the soaking experiments. JP also carried out the X-ray diffraction, data processing, and structure refinement, as well as the ITC experiments. JP drafted the manuscript and constructed the figures. Y-SL synthesized the compound YS0470. Y-SL and JP together purified and crystallized the human FPPS protein. JWDS carried out the DSF experiments. YST developed the structure-guided approach used to design the compound YS0470 and helped revise the manuscript. AMB participated in analyzing the X-ray diffraction data and drafting the manuscript. All authors read and approved the final manuscript.

## Supplementary Material

Additional file 1 ** Table S1.** Average B-factors for the overall structure and the four C-terminal residues of human FPPS complexes. Click here for file

Additional file 2 ** Figure S1.** Electron density maps and average B-factors for the residues involved in the human FPPS tail closure. The structure of the Pi-bound complex is represented in green, and that of the PPi-bound complex in cyan. The 2*F*_o_-*F*_c_ maps for the residues of interest are contoured at 1.0 sigma level and shown in respective colors. The average B-factor for each residue was calculated only for the side chain. The overall B-factors of the two structures are very similar (Additional file 1: Table S1). Click here for file
