# Supplementary material for: Ternary complex structures of human farnesyl pyrophosphate synthase bound with a novel inhibitor and secondary ligands provide insights into the molecular details of the enzyme’s active site closure
Source: BMC Struct Biol. 2012 Dec 12;12:32. doi: 10.1186/1472-6807-12-32 (PMC3539973; doi:10.1186/1472-6807-12-32)
Supplement: Additional file 1 — Table S1. Average B-factors for the overall structure and the four C-terminal residues of human FPPS complexes. [file 1472-6807-12-32-S1.pdf]

**Human FPPS Structure**	**Overall average B-factor (Å^2)**	**Average B-factor (Å^2) ^{350}KRRK^{353} tail**
Pi-bound complex [PDB: 4H5C]	41.2	85.7
PPi-bound complex [PDB: 4H5D]	40.7	47.4
IPP-bound complex [PDB: 4H5E]	44.8	52.9
