# Supplementary figures and images for: Ternary complex structures of human farnesyl pyrophosphate synthase bound with a novel inhibitor and secondary ligands provide insights into the molecular details of the enzyme’s active site closure
Source: BMC Struct Biol. 2012 Dec 12;12:32. doi: 10.1186/1472-6807-12-32 (PMC3539973; doi:10.1186/1472-6807-12-32)

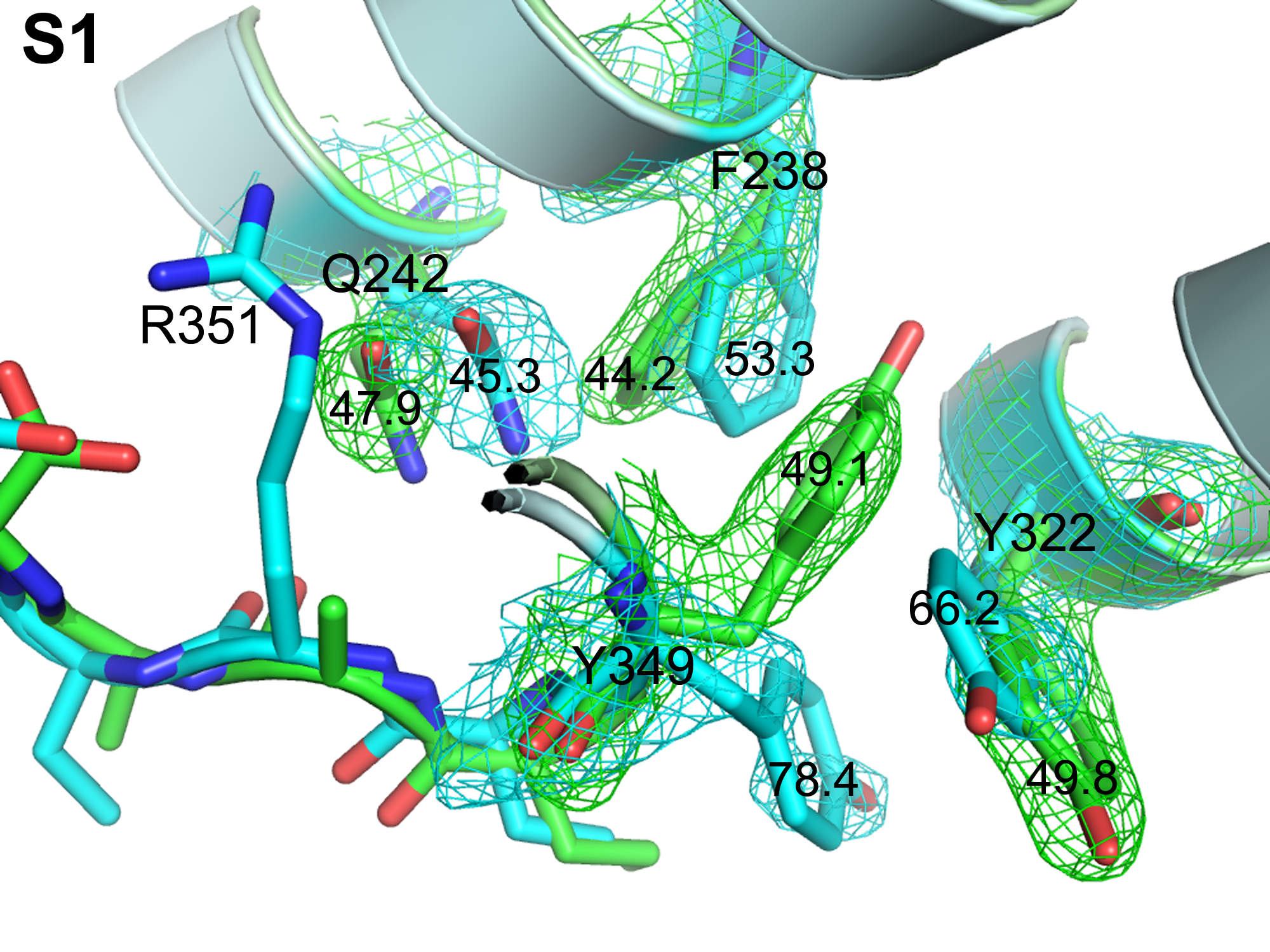

Supplement: Additional file 2 — Figure S1. Electron density maps and average B-factors for the residues involved in the human FPPS tail closure. The structure of the Pi-bound complex is represented in green, and that of the PPi-bound complex in cyan. The 2Fo-Fc maps for the residues of interest are contoured at 1.0 sigma level and shown in respective colors. The average B-factor for each residue was calculated only for the side chain. The overall B-factors of the two structures are very similar (Additional file 1: Table S1). [file 1472-6807-12-32-S2.tiff]
